# Sustained hypoxia but not intermittent hypoxia induces HIF-1α transcriptional response in human aortic endothelial cells†

**DOI:** 10.1039/d4mo00142g

**Published:** 2024-10-22

**Authors:** Rengul Cetin-Atalay, Angelo Y. Meliton, Yufeng Tian, Kaitlyn A. Sun, Parker S. Woods, Kun Woo D. Shin, Takugo Cho, Alex Gileles-Hillel, Robert B Hamanaka, Gökhan M. Mutlu

**Affiliations:** a Department of Medicine, Section of Pulmonary and Critical Care Medicine, University of Chicago Chicago IL USA rengul@uchicago.edu gmutlu@uchicago.edu; b Department of Pediatrics, Hadassah Medical Center, Hebrew University Jerusalem Israel

## Abstract

Obstructive sleep apnea (OSA) is characterized by intermittent hypoxic environments at the cellular level and is an independent risk factor for the development of cardiovascular disease. Endothelial cell (EC) dysfunction precedes the development of cardiovascular disease; however, the mechanisms by which ECs respond to these intermittent hypoxic events are poorly understood. To better understand EC responses to hypoxia, we examined the effects of sustained hypoxia (SH) and intermittent hypoxia (IH) on the activation of HIF-1α in ECs. While SH stabilized HIF-1α and led to its nuclear localization, IH did not activate HIF-1α and the expression of its target genes. Using RNA-sequencing, we evaluated transcriptional responses of ECs to hypoxia. SH induced the expression of HIF-1α and hypoxia response genes, while IH affected cell-cycle regulation genes. A cytoscape protein–protein interaction network for EC response to hypoxia was created with differentially expressed genes. The network comprises cell-cycle regulation, inflammatory signaling *via* NF-κB and response to VEGF stimulus subnetworks on which SH and IH had distinct activities. As OSA is associated with elevated catecholamines, we investigated the effect of epinephrine on the EC response to SH and IH. Transcriptomic responses under IH and epinephrine revealed protein–protein interaction networks emphasizing distinct subnetworks, including cytokine-mediated TNFα signaling *via* NF-κB, Wnt/LRP/DKK signaling and cell cycle regulation. This study reveals differential transcriptomic responses under SH and IH characterised by HIF-1α transcriptional response induced only by SH, but not by IH. The study also features the potential molecular events that may occur at the vascular level in OSA.

## Abstract 2

This study investigates how endothelial cells respond to sustained (SH) and intermittent (IH) hypoxia, a hallmark of obstructive sleep apnea. HIF-1α was induced by SH but not by IH. SH and IH activate distinct transcriptional responses.

## Graphical abstract

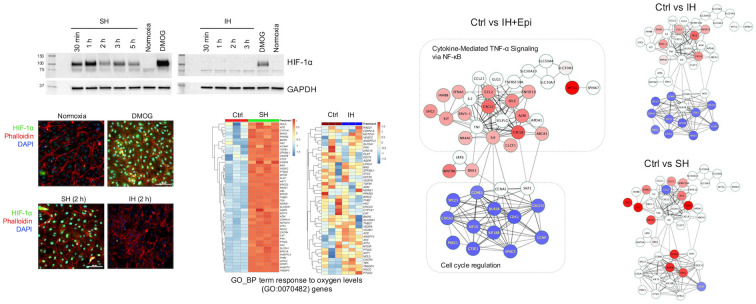

## 1. Introduction

Obstructive sleep apnea (OSA) is characterized by repetitive episodes of partial or complete obstruction of the upper airway during sleep, leading to partial (hypopneas) or complete (apneas) reductions in airflow, respectively. These episodes result in a cycle of hypoxic microenvironment at the cellular level, where tissues are intermittently exposed to low oxygen levels.^1,2^ This recurrent hypoxia triggers various cellular and systemic level responses such as catecholamine release, potentially contributing to the development of a range of pathological conditions including cardiovascular disease.^1,3^ Our understanding of OSA and its relationship to cardiovascular disease has made considerable progress; however, the detailed mechanisms by which OSA increases cardiovascular risk are not fully elucidated.^4^ OSA is known to be linked with dysfunction and activation of endothelial cells (ECs), the development of which precedes the onset of cardiovascular diseases.^1,5–7^ Despite this association, the specific effects of varying hypoxic conditions on EC function remain poorly understood. Hypoxia-inducible factor 1-alpha (HIF-1α) functions as a principal transcriptional regulator of the adaptive response to hypoxia across various pathological conditions.^8^ During normal oxygen levels (normoxia), oxygen-dependent proline hydroxylases target HIF-1α for proteasomal degradation. However, under low oxygen conditions (hypoxia), decreased activity of these hydroxylases leads to the stabilization, nuclear translocation and transcriptional activation of HIF-1α protein. In vascular health, this oxygen-dependent adaptive response can have significant implications, as HIF-1α activation leads to the transcription of genes that facilitate both survival under reduced oxygen conditions and angiogenesis. Under hypoxic conditions, over 300 HIF-1α target genes are differentially expressed. These genes are involved in energy metabolism, angiogenesis, apoptosis, and increase in oxygen delivery or facilitate metabolic adaptation to hypoxia.^9^ Understanding how HIF-1α is stabilized and functions in different hypoxic scenarios is essential for deciphering the cellular behaviors that contribute to the pathology and progression of vascular diseases. Despite the wealth of knowledge gained in recent years about cellular responses to hypoxia, it remains incompletely understood whether cells respond to IH with similar transcriptional mechanisms and programs. Discrepant findings exist in the literature, indicating the complexity of the relationship between hypoxia induced by OSA and EC response due to variations in the severity and duration of hypoxia used in models to mimic IH.^7,10–15^

Recently, our group and others have used a short duration of intermittent hypoxic culture medium, which more accurately reflects the hallmark features of OSA-related hypoxic pathophysiology to unravel the mechanisms by which OSA is associated with the dysfunction and activation of ECs. We showed that IH induces pro-inflammatory adhesion, leukocyte migration, angiogenesis, and extracellular matrix organization genes in ECs, similar to what is found in ECs *in vivo*.^7,14^

Here, we conducted a comparative transcriptomic analysis to examine the effects of sustained hypoxia (SH) and IH on HIF-1α protein stabilization and its downstream gene expression in human aortic endothelial cells (HAECs). We found that IH does not stabilize HIF-1α protein unlike SH, which activates HIF-1α and its downstream pathways. Our transcriptional analysis indicates distinct gene expression patterns under IH compared to both normoxia and SH. While SH predominantly influences genes related to oxygen sensing, protein stability, and cellular proliferation, IH impacts genes involved in DNA replication and sterol metabolism. Additionally, we explored the interactions between intermittent hypoxic stress and adrenergic signaling in HAECs and found that adrenergic signaling activates genes associated with HIF-1α. This comprehensive investigation provides valuable insights into the cellular mechanisms activated by different hypoxic conditions, enhancing our understanding of the cellular adaptations characteristic of OSA, particularly those related to IH and sympathetic activation.

## 2. Materials and methods

### 2.1. Cells

We used primary HAECs in our studies. HAECs were purchased from Lonza (Allendale, NJ) (CC-2535, lot number 35034, 35998, or 40830). Cells were grown in EGM-2 supplemented with SingleQuots from Lonza (CC-3156 and CC-4176) and penicillin/streptomycin/amphotericin B solution (Sigma). Epinephrine hydrochloride (Sigma) was solubilized in water and kept in −20 °C in aliquots of 10 mM of concentration. All primary cultures were used from passage 5 to 7.

### 2.2. *In vitro* exposure of HAECs to sustained hypoxia or intermittent hypoxia

HAECs were exposed to sustained hypoxia (SH) (1% O_2_) in HypOxystation H35 chamber (HypOxygen, USA) for 6 hours or intermittent hypoxia (IH) exposure for 60 cycles (1% O_2_ for 2 minutes alternating with 20% O_2_ for 5 minutes). Exposure to IH was achieved by two independent methods. Media for both hypoxic and normoxic conditions were prepared by infusing the respective gases (1% or 20% O_2_, 5% CO_2_ and N_2_) prior to the experiments and O_2_ levels were regularly monitored by dissolved oxygen meter (Orion, Thermo Scientific). The CO_2_ level for both control normoxia and IH exposure was kept at 5% with nitrogen for the balance. We used a cyclic perfusion system with a flow rate set at 5 mL min^−1^. Concurrently, HAECs were subjected to either normoxic (control) or hypoxic culture media (IH). The control group had similar flow conditions as the IH group, using the same perfusion system. Each circuit, hypoxic and normoxic, was equipped with its own pump to maintain an intermittent yet constant flow of conditioned media in a four-chamber interconnected culture dish. Second intermittent hypoxia exposure was achieved by using a pump system that alternately flushed normoxic (20% O_2_ for 5 minutes) medium and hypoxic medium (1% O_2_ for 2 minutes) into the culture from the side of the dish, and then aspirated and replaced the media. The entire setup for both systems was maintained at a temperature of 37 °C in a cell culture incubator.

### 2.3. Western blot

Cells were lysed in 100 μL urea sample buffer (8 M deionized urea, 1% SDS, 10% glycerol, 60 mM Tris pH 6.8, 0.1% pyronin-Y, 5% 2-mercaptoethanol) as we previously described (Robs Paper). Lysates were run through a 28-gauge needle and were electrophoresed on criterion gels (Bio-Rad) and transferred to nitrocellulose using a Trans-Blot Turbo (Bio-Rad) set to the mixed MW program. Membranes were blotted with HIF-1α (Cayman Chemicals 10006421) antibodies.

### 2.4. Immunofluorescence staining

Cells were plated onto glass slides, and immunofluorescence staining was performed as described in Regev *et al.*^16^ At the end of the exposure to SH or IH, cells were washed twice with cold phosphate-buffered saline (PBS) and then fixed with 4% paraformaldehyde. The cells were permeabilized and blocked using a solution containing 0.1% Triton X100 and 3% fetal bovine serum (FBS) in PBS for 60 minutes. They were then washed with PBS and incubated with HIF-1α primary antibody (Abcam ab179483, diluted 1 : 100 in 3% FBS). After a subsequent washing step, the cells were incubated with secondary antibodies, specifically CoraLite®488-Conjugated AffiniPure. Concurrently, CoraLite®594-conjugated phalloidin antibody was added to stain the actin cytoskeleton. Post staining, the slides were washed and mounted with ProLong™ NucBlue™ Stain. Imaging was performed using an ECLIPSE Ti2 inverted microscope and NIS software (NIKON, USA).

### 2.5. Quantitative PCR

RNA was isolated GenElute™ Mammalian Total RNA Miniprep Kit and reverse transcribed using iScript Reverse Transcription Supermix (Bio-Rad, catalog number 1708841). Quantitative mRNA expression was determined by real-time quantitative PCR (qPCR) using iTaq Universal SYBR Green Supermix (Bio-Rad, catalog number 172-5121). RPL19 was used as a housekeeping gene, and gene expression was quantified using the 2^ΔΔCt^ method to determine relative fold-change in 3 biological 3 technical replicates. Primer sequences that were designed to span exon junctions are as follows: *RPL19* (5′-AGTATGCTCAGGCTTCAGAAGA-3′, 5′-CATTGGTCTCATTGGGGTCTAAC-3′), *HIF1A* (5′-GGCGCGAACGACAAGAAAAA-3′, 5′-GTGGCAACTGATGAGCAAGC-3′), *HK2* (5′-GCTTGGAGCCACCACTCACCC-3′, 5′-AGCCAGGAACTCTCCGTGTTCTGT-3′), *EGLN3* (5′-CACAGCGAGGGAAT GAACCT-3′, 5′-TCCTGCTGTTAAGGCTTCCG-3′).

### 2.6. RNA isolation and sequencing

Total RNA was extracted from HAECs with the GenElute™ Mammalian Total RNA Miniprep Kit. RNA quality was evaluated with Bioanalyzer (Agilent), and RIN values were >8.5. RNA was submitted for sequencing at the University of Chicago Genomics Core Facility, Illumina NovaSEQ6000 sequencer (paired-end). FASTQ files, which underwent quality assessment per base sequence through FastQC. RNA-seq reads were pseudoaligned using Kallisto v.0.44.0 the at Center for Research Informatics, Randi high performance computing cluster, University of Chicago.^17^ The Kallisto index was made with default settings utilizing GENCODE (Release 39 (GRCh38.p13)) and quantification was executed in its default mode. Gene abundance calculations were performed with the tximport R package v.1.18.03, and differential expression analysis between normoxic and hypoxic conditions was conducted using the EdgeR R package, which involves read count filtering, normalization, dispersion estimation, and the identification of differentially expressed genes.^18^ Significance in differential gene expression was attributed to genes with an FDR-adjusted *p*-value ≤0.05 and a fold change (FC) > 2. Visualization of results included volcano plots *via* ggplot2 R package and heatmaps using the pheatmap package from Z-score normalized expression data. Pathway enrichment analyses were performed, and plots were created using R clusterProfiler package^19^ or using Enrichr search engine web interface on Enrichr database 35.^20^ For HIF-1α target gene set enrichment ChEA3 (Chip-X Enrichment Analysis Version 3) were used which collects information on TF/target-gene interactions from a variety of experimental assays and additional evidence sources. It ranks TFs by comparing the user-provided gene groups with the ChEA3 database's catalogued collections of TF targets. All packages were run on RStudio (2023.12.1, build402) with R version 4.3.2. Transcription factor (TF) target gene interactions gene regulatory network enrichment analysis was done with R DoRothEA package using human regulons.^21^ Protein–protein interaction networks were visualized with Cytoscape software platform and stringApp.^22,23^ Protein–protein interaction data was imported from String-db.^24^ RNA-seq data is accessible *via* GEO (GSE205050) and (GSE279434).

### 2.7. Statistics

The qPCR data from 3 independent biological replica were analyzed in Prism 10 (GraphPad Software Inc., La Jolla, CA). All data are shown as mean ± SEM. Significance was determined by unpaired, two-tailed Students *t*-test (for comparisons between two samples) or by one-way ANOVA using Bonferronis correction for multiple comparisons. Statistical significance was defined as *p < 0.05, **p < 0.005, ***p < 0.001, ****p < 0.0001.

## 3. Results

### 3.1. The effect of sustained hypoxia and intermittent hypoxia on HIF-1α protein stabilization and target gene expression in HAECs

**Fig. 1:**
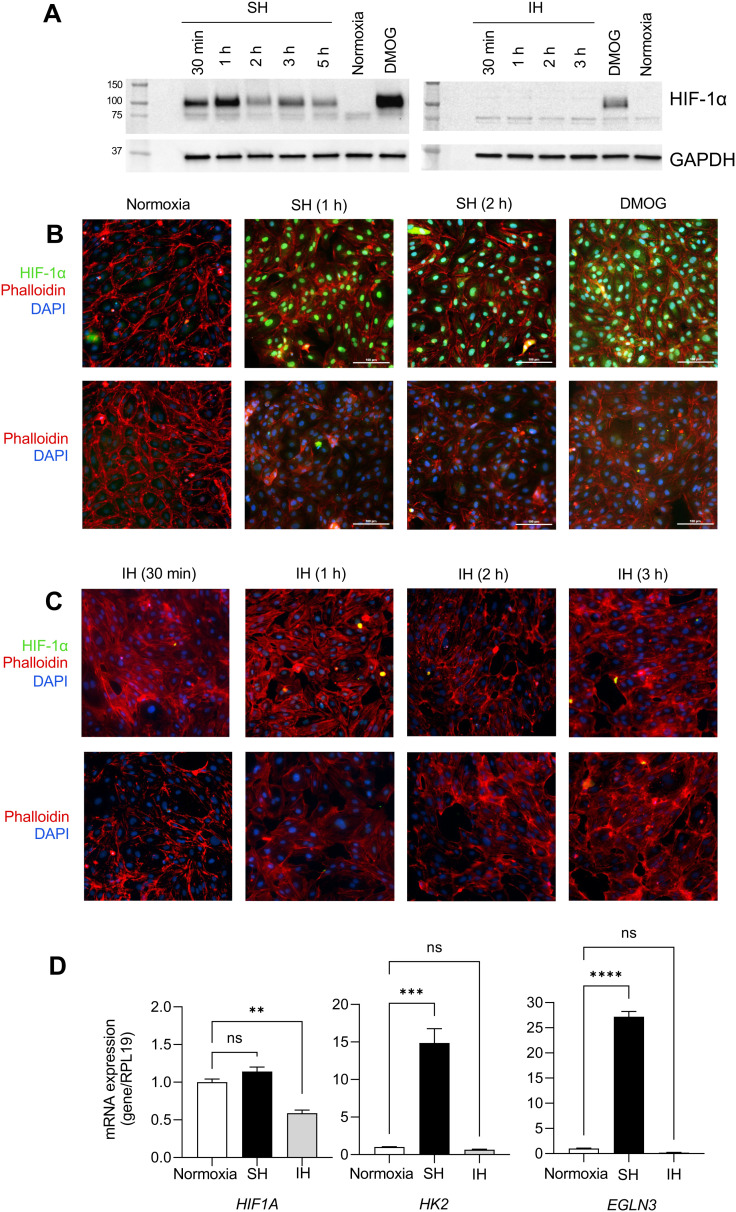
Human aortic endothelial cells display HIF-1α stabilization and increased expression of downstream HIF-1α target genes under sustained hypoxia but not intermittent hypoxia. (A) HAECs were incubated under sustained hypoxia (SH) or intermittent hypoxia (IH) for indicated times. Western blot analyses of total cellular extract were performed to assess HIF-1α protein stabilization. Dimethyloxalylglycine (DMOG) was used as a positive control. (B) and (C) Immunofluorescence images demonstrating nuclear expression of HIF-1α protein (green) in HAECs under SH at indicated times. HIF-1α protein was not expressed in nuclei under IH. DAPI (blue) and phalloidin (red) stains are used for nuclear and actin cytoskeleton labeling, respectively. (D) mRNA expression levels of *HIF1A* and downstream target genes of HIF-1α, *HK2*, and *EGLN3* were measured in HAECs under 6 hours of SH or IH by qPCR (data are shown as mean ± SEM, *n* = 3, *p < 0.05).

To determine the effect of IH on HIF-1α, we exposed HAECs to SH or IH and collected samples starting from 30 min of exposure. At the end of exposure, we measured the expression of HIF-1α protein by western blotting and the expression of HIF-1α target genes by qPCR. HAECs exposed to SH showed significant stabilization of HIF-1α protein in total cellular extracts at all time points (Fig. 1A and Fig. S1, ESI†).

In contrast, IH failed to induce the stabilization of HIF-1α protein in HAECs. We utilized dimethyloxalylglycine (DMOG), which is a competitive inhibitor of prolyl-4-hydroxylase domain (PHD) enzymes that regulate the stability of HIF-1α, as a positive control for HIF-1α expression. Complementing these findings, immunofluorescence staining revealed the nuclear localization of HIF-1α protein in HAECs under SH (Fig. 1B). In contrast, and similar to the lack of induction of HIF-1α protein expression, IH did not induce nuclear stabilization of HIF-1α protein (Fig. 1C). *EGLN1* and *EGLN3* are the regulators of HIF-1α under normoxia, while *EGLN3* expression is induced upon HIF-1α activation, makes this gene the most important isozyme in limiting physiological activation of HIF-1α in hypoxia.^11,25,26^ When we examined the mRNA expression levels of HIFA and the well-known HIF-1α target genes, *HK2*^27^ and *EGLN3*^26^ using qPCR. Consistent with increased HIF-1α protein stabilization, SH increased the expression of *HK2* and *EGLN3* mRNA; however, we did not observe any significant changes in the expression of these genes in HAECs exposed to IH (Fig. 1D) collectively, our results distinctly highlight the differential response of HAECs to SH, which induces the expression of HIF-1α and its target genes and IH, which fails to induce HIF-1α expression.

### 3.2. Differential transcriptional responses of HAECs to SH and IH

**Fig. 2:**
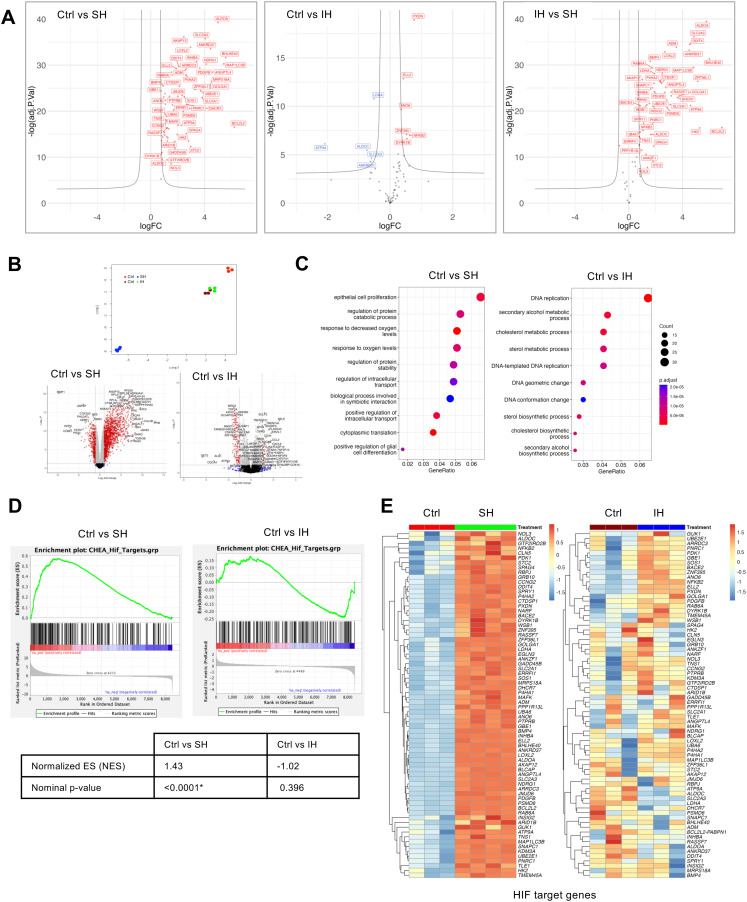
Comparative transcriptional analysis in human aortic endothelial cells under sustained or intermittent hypoxia. We exposed HAECs to either normoxia control (Ctrl) or SH or IH for 6 hours. RNA-sequencing was performed on total RNA extracted from cells at the end of exposure. (A) Volcano plot of HIF-1α target genes from the CHEA transcription factor targets dataset (total of 314 genes) in Ctrl *vs*. SH showing 70 significantly differentially expressed under SH. Volcano plots of these 70 genes were shown for Ctrl *vs*. IH, and SH *vs*. IH as comparisons. (B) The differential mRNA expression similarities of HAECs under SH or IH compared to Ctrl were visualized by Uniform Manifold Approximation and Projection (UMAP) of log 2 fold changes (log FC) (*n*-neighbors = 4, *n*-components = 3) and volcano plots of 8425 DEGs. (C) Top ten significant GO-BP terms upon GO Enrichment Analysis (R cluster profiler “enrichGO” function) of gene sets from HAECs under SH or IH compared to Ctrl (*p*-value cutoff = 0.01 with Bonferroni adjustment method). (D) Comparisons of gene set enrichment plots and (E) heatmaps of CHEA HIF-1α targets in HAECs under SH or IH relative to Ctrl. The criteria for determining the significance of differentially expressed genes (DEGs) were set to an absolute fold change of ≥2 and an FDR-adjusted *p*-value of ≤0.05.

RNA sequencing was utilized to examine the impact of these conditions on the transcriptome, aiming to better understand the differential effects of SH and IH on HAECs. We found that SH caused distinct patterns of gene expression consistent with HIF-1α activation compared to normoxic controls as expected, while IH had a smaller effect on gene expression in general (Fig. S2, ESI†). We obtained HIF-1α transcription factor (TF) targets from The CHEA Transcription Factor Targets dataset, which included data on 314 HIF-1α target genes identified through multiple low- and high-throughput assays of HIF-1α transcription factor activity.^9,28^ Seventy genes out of 314 HIF-1α target genes were significantly differentially expressed in HAECs under SH when compared to Ctrl. The volcano plot analysis of the differentially expressed HIF-1α target genes showed significant upregulation of genes under SH when compared to control or IH (Fig. 2A).

Uniform manifold approximation and projection (UMAP) dimensionality reduction and Volcano plots of differentially expressed genes (8425 DEGs) highlighted the gene expression similarities between normoxia controls and SH or IH, demonstrating distinct transcriptional responses to SH with respect to normoxic controls and IH (Fig. 2B) Gene Ontology Biological Process (GO-BP) enrichment analysis of gene sets from HAECs under SH identified HIF-1α associated GO-BP terms such as response to oxygen levels and protein stability and cell proliferation while DNA replication and cholesterol/sterol metabolic process GO-BP terms were affected by IH (Fig. 2C). We also performed a custom gene set enrichment analysis (GSEA) with the 314 ChEA3 HIF-1α target gene set on all differentially expressed genes from SH and IH samples (Fig. 2D). Enrichment plots of Ctrl *vs*. SH and Ctrl *vs*. IH and normalized enrichment scores of 1.43 and −1.02 and *p* values of <0.0001 and 0.396 respectively, demonstrated the HIF-1α activation dependent transcriptional activity under SH but not IH. Heatmaps further detailed the impact of SH and IH on significantly affected HIF-1α target genes (Fig. 2E). Our comprehensive transcriptional analysis delineates the response of HAECs to SH and IH compared with the normoxic controls providing insight into the regulatory mechanisms influenced by different hypoxic conditions.

### 3.3. Distinct endothelial cell and oxygen responses in HAECs under IH and SH conditions

**Fig. 3:**
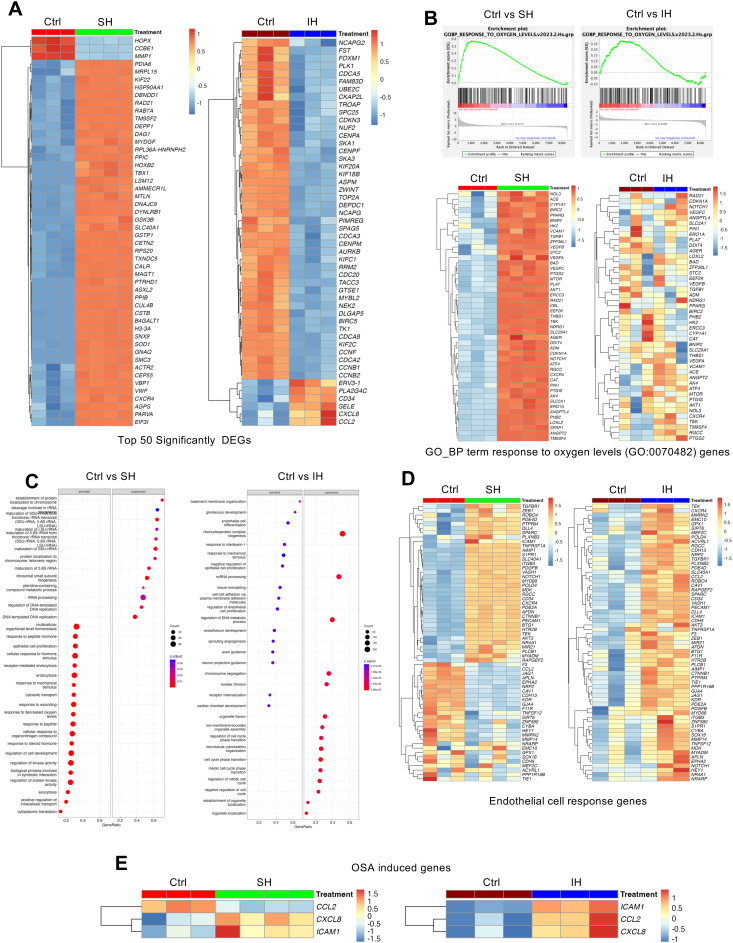
Differential response of human aortic endothelial cells to sustained and intermittent hypoxia. Differentially expressed gene profile analysis of HAECs under normoxia (Ctrl) was compared to SH or IH. (A) Heatmap of top 50 significantly differentially regulated genes. (B) GO-BP response to oxygen levels (GO:0070482) gene set enrichment plots and heat maps HAECs under SH or IH relative to normoxia (Ctrl). (C) Activated and suppressed GO-BP terms, based on gene set enrichment analysis (R cluster profiler “gseGO” function) (*p* ≤ 0.05), under SH or IH demonstrating endothelial cell responses. (D) Heatmap of endothelial cell response gene list from the GO-BP terms obtained with gseGO (gene list given in the ESI†). (E) Heat map of three co-occurring genes (*CCL2*, *CXCL8*, and *ICAM1*) with the biological term ‘osasinduced’ in literature-supported statements that describe the functions of genes sourced from the NLM GeneRIF Biological Term Annotations dataset (https://www.ncbi.nlm.nih.gov/gene/about-generif).

Differential expression profiles analysis of HAECs exposed to SH and IH revealed different gene expression profiles as highlighted by the heat map of the top 50 significantly regulated genes compared with normoxic controls (Ctrl) (Fig. 3A).

GSEA of the GO-BP term response to oxygen levels (GO:0070482) gene set indicated a distinct pattern of differentially expressed genes from both hypoxic conditions (Fig. 3B). Heat map of the GO:0070482 gene set demonstrated individual EC responses with respect to different hypoxic conditions (Fig. 3B). While the genes of the GO:0070482 gene set were uniformly upregulated under SH, the expression of these genes were highly variable under IH. Furthermore, SH and IH had different effects on the expressions of GO-BP *endothelial cell response genes* term gene set (Fig. 3D) suggesting the complexity of cellular response to different types of hypoxic conditions. Lastly, we examined the expression of genes (*CCL2*, *CXCL8*, *ICAM1*) from ‘osasinduced’ NIH GeneRIF Biological Term Annotations. We found that induction of all three genes were significantly induced by IH. *CXCL8* and *ICAM1* were induced to a lesser extent under SH and expression of *CCL2* was decreased (Fig. 3E).

**Fig. 4:**
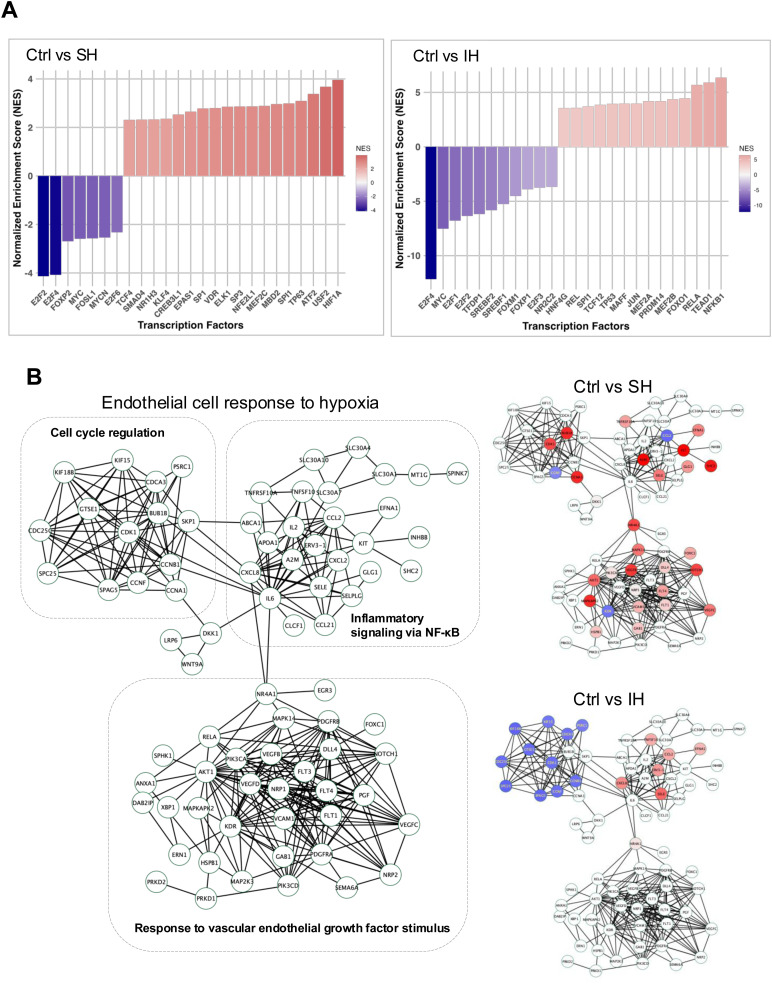
Hypoxia dependent gene regulatory signaling in HAECs. (A) Transcription factor enrichment was computed using the DoRothEA R tool with significantly differentially expressed genes (log FC ≤ 0.5, *p* ≤ 0.05) for the following comparisons: control normoxia (Ctrl) *vs*. SH or Ctrl *vs*. IH. The color legend indicates TF activity. (B) The Cytoscape software platform was used to construct a protein–protein interaction network of endothelial cell response to hypoxia, edges imported from the STRING database. Comparative visualization of downstream gene expression profiles Ctrl *vs*. SH, Ctrl *vs*. IH are visualized based on their expression values, genes with increased expression are marked in red, while those with decreased expression are represented in blue.

Based on the differential transcriptional responses of HAECs to SH and IH, we constructed a protein–protein interaction (PPI) network using the STRING database with a gene list derived from GO-BP terms *response to oxygen levels* and *endothelial cell response*, and visualized on the Cytoscape software platform (network .cys files are provided as ESI†).^23,24^ The network (78 protein nodes and 312 PPI edges) comprised three distinct subnetwork modules which involves genes in cell cycle regulation, inflammatory signaling *via* NF-κB and response to vascular endothelial growth factor stimulus (Fig. 4B).

We then mapped significant DEGs from the responses in HAECs to SH and IH treatments compared with normoxic control (Fig. 4B, Ctrl *vs*. SH and Ctrl *vs*. IH). Notably, while SH altered the expression of genes in all three network modules, the DEGs of IH mapped only on cell cycle regulation, inflammatory signaling *via* NF-κB network modules.

### 3.4. The effect of epinephrine on HIF-1α stabilization and hypoxia-associated genes

**Fig. 5:**
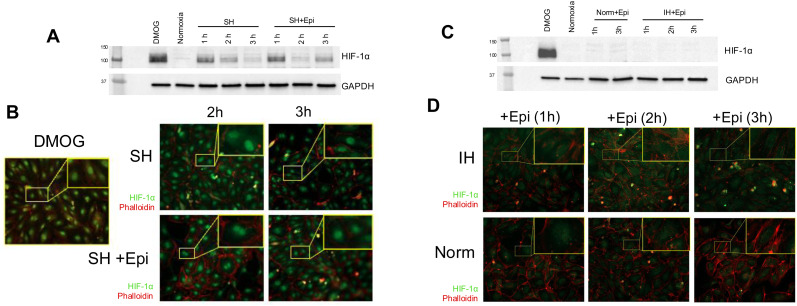
Epinephrine treatment stabilizes HIF-1α protein under sustained hypoxia in HAECs. (A) and (B) HAECs were treated at indicated times with 10 μM epinephrine (Epi) under sustained hypoxia (SH) or normoxia (Norm). HIF-1α protein stabilized and localized to nucleus under sustained hypoxia in the presence of epinephrine. Western blot and immunofluorescent images demonstrating the expression of nuclear HIF-1α protein (green) under SH at indicated times in HAECs. (C) and (D) There is a minor HIF-1α protein stabilization and nuclear localization of HIF-1α protein in HAECs treated with epinephrine (Epi) under normoxia which is lost under intermittent hypoxia (IH). DAPI (blue) and phalloidin (red) stains are used as nuclear and actin cytoskeleton labelling, respectively. Dimethyloxalylglycine (DMOG) was used as a positive control.

We have previously shown that IH-induced EC activation *in vivo* is partially mediated *via* catecholamines through sympathetic nervous system activation.^5,6^ In this study, we analyzed HAECs treated with 10 μM epinephrine under SH and IH conditions. SH displayed significant stabilization and nuclear localization of HIF-1α protein in HAECs (Fig. 5A and B).

**Fig. 6:**
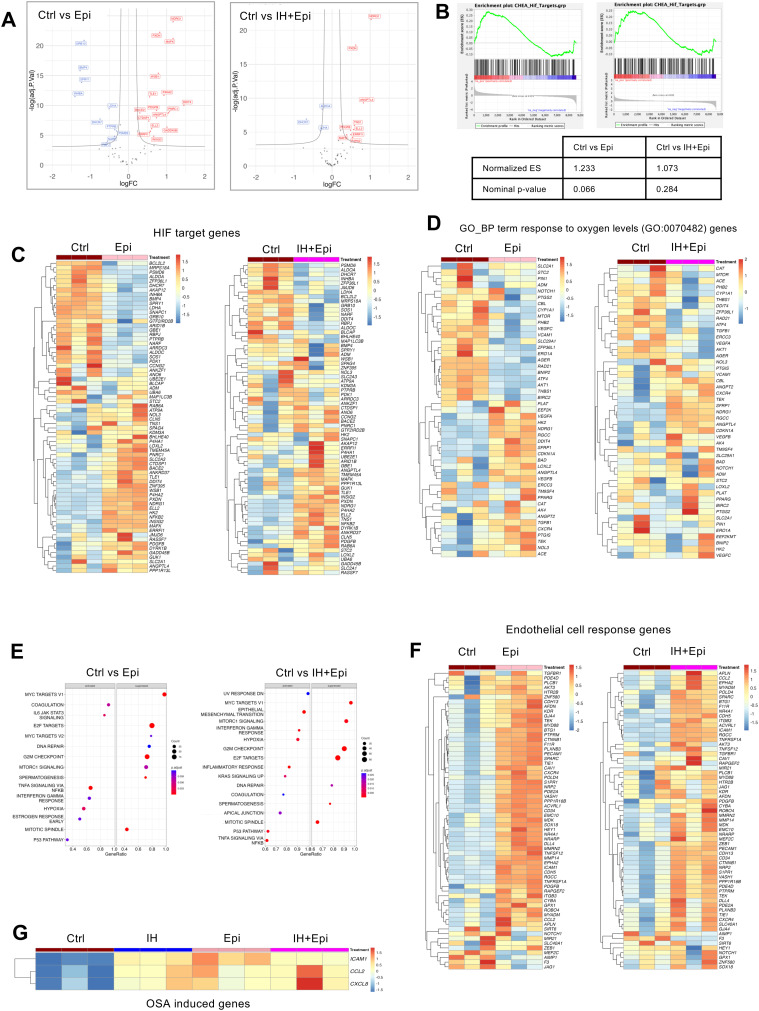
Hypoxia dependent gene regulatory signaling in HAECs. Effect of epinephrine in HIF-1α target gene and hypoxia-associated gene expression responses in human aortic endothelial cells. We treated HAECs with either epinephrine (Epi) (10 μM) or vehicle control under normoxia (Ctrl) or IH. We then performed RNA-sequencing in RNA isolated from cells. (A) Volcano plots, (B) gene set enrichment plots, and heatmaps of (C) CHEA HIF-1α target genes and (D) heatmaps of GO-BP response to oxygen levels (GO:0070482) genes following treatment with epinephrine under control normoxia or IH. (E) Activated and suppressed GO-BP terms were identified by gene set enrichment analysis of DEGs in HAECs treated with epinephrine under control normoxia or IH. (F) Heatmaps of GO-BP term endothelial cell response following treatment with epinephrine under control normoxia or IH. (G) Comparative expression heatmap of OSA-induced genes (*CCL2*, *CXCL8*, and *ICAM1*) in control, IH, and epinephrine alone or with IH.

However, only minor stabilization and nuclear localization of HIF-1α was observed in HAECs that were treated with epinephrine under normoxia, the effect of epinephrine on HIF-1α was lost under IH (Fig. 5C and D). We also analyzed the transcriptome level effect of epinephrine with respect to HIF-1α target gene expression and hypoxia-associated gene sets as well as EC response gene expression (Fig. 6).

Volcano plots showed an increased number of genes regulated by epinephrine (Fig. 6A) compared to IH alone (Fig. 2A). CHEA_Hif_Target gene set enrichment plot of Ctrl *vs*. Epi and its normalized enrichment score of 1.23 and *p* value of 0.066 suggested a trend towards hypoxia-independent HIF-1α activation under epinephrine alone (Fig. 6B, left panel). In contrast, Ctrl *vs*. IH + Epi suggested that IH reduced the effect of epinephrine on HIF-1α activation (Fig. 6B, right panel). Heat maps further confirmed the effect of epinephrine on CHEA_Hif_Target gene enrichment analysis under normoxia and IH (Fig. 6C and D). Heat map of the GO:0070482 gene set demonstrated distinct EC responses to epinephrine and IH + epinephrine (Fig. 6D). Gene set enrichment analysis revealed differential effects of IH and IH + Epi on EC gene expression, highlighting the influence of epinephrine on the EC response to IH (Fig. 6E and F). Additionally, the expression of “OSA-induced” genes (*CCL2*, *CXCL8*, *ICAM1*) was increased not only under IH but also epinephrine alone (Fig. 6G). We also performed DoRothEA TF enrichment analysis, under epinephrine treatment under normoxia. *PRDM14*, *RELA*, *NFKB1*, *CREM*, and KLDs (*KLF1*, *5*, *6*, *9*) were TFs involved in gene expression regulation (Fig. S3, ESI†). TFs under IH and epinephrine were similar to those under IH. *HIF1A* was also identified under IH and epinephrine. Significant DEGs from epinephrine treatments were mapped to the PPI network in Fig. 4B (Fig. S3, ESI†). Epinephrine treatment under normoxia alter the expression of genes in all three network modules, whereas IH with epinephrine does not alter the expression of genes involved in the response to vascular endothelial growth factor stimulus network module (Fig. S3, ESI†). Epinephrine alone or IH with epinephrine downregulates cell cycle regulation genes which are cyclins, CDKs and kinesin family of genes. Genes involved in inflammatory signaling *via* NF-κB network module were upregulated under IH with epinephrine.

## 4. Discussion

Obstructive sleep apnea is a common sleep disorder marked by repetitive episodes of complete or partial blockages of the upper airway while asleep. OSA is a significant risk factor for cardiovascular diseases.^2–4^ The pathophysiological hallmark of OSA, IH results from the cyclic cessation of airflow and is associated with systemic inflammation, and sympathetic activation. The repeated episodes of low oxygen levels (hypoxia) and reoxygenation stimulate both the sympathetic activity and the release of cytokines, which play a key role in the inflammatory response in ECs.^1^ We and others have shown that IH induces the release of cytokines, including tumor necrosis factor-alpha (TNFα) and interleukin-6 (IL-6) leading to EC activation.^5,6,14^ Current available studies on the effects of IH on EC responses demonstrate considerable variability, influenced by the duration and severity of pericellular hypoxia affected by experimental conditions used to mimic OSA.^7,13^ Here, we show that despite the involvement of EC activation genes associated with inflammation and angiogenesis, short episodes of IH do not activate the critical hypoxia dependent transcription factor, HIF-1α, indicating the intricate nature of how SH and IH affect ECs and induce SH and IH specific response. Our research investigated the complex interplay between hypoxia and ECs under SH and IH on the stabilization and transcriptional activation of HIF-1α. SH robustly stabilizes HIF-1α protein, leading to substantial increases in both protein levels and nuclear localization, as evidenced by our western blotting and immunofluorescence staining results. This stabilization correlates with an upregulation of key HIF-1α target genes such as *HK2* and *EGLN3*, reflecting a traditional hypoxic response that enhances cellular adaptation to low oxygen conditions. In clear contrast, IH does not yield a similar effect, showing no significant stabilization of HIF-1α protein or induction of its nuclear localization. Furthermore, our transcriptional analysis strengthens the understanding of EC responses toward different hypoxic conditions. The differential expression profile under SH, as confirmed by GO enrichment and custom gene set enrichment analyses aligns with the expected activation of HIF-1α-mediated pathways involved in cellular responses to hypoxia. Conversely, IH had no impact on the expression of HIF-1α target genes, suggesting that the transient nature of IH might not sustain the signals necessary for HIF-1α activation.

**Fig. 7:**
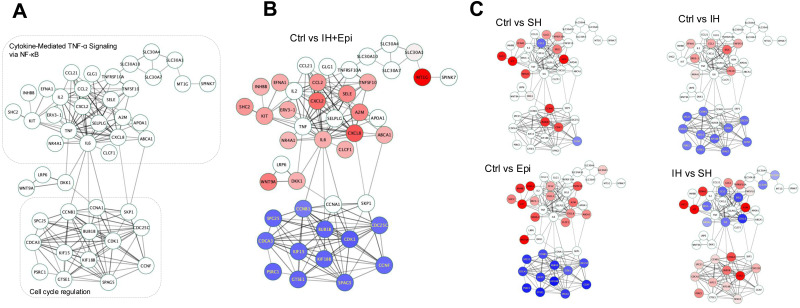
OSA associated Endothelial cell response protein–protein interaction network. (A) We have constructed and curated a new protein–protein interaction network by applying thresholds of |log FC| ≥ 1.5 and *p*-value <0.05 to transcriptome expression data from HAECs subjected to IH and treated with epinephrine. This novel network comprises 44 protein nodes and 148 interaction edges, derived from STRING database. Gene expression profiles comparing (B) Ctrl *vs*. IH + Epi, (C) Ctrl *vs*. IH, Ctrl *vs*. CH, Ctrl *vs*. Epi, and IH *vs*. SH and were mapped onto this novel network. Genes exhibiting up-regulation are indicated in red, while those showing down-regulation are highlighted in blue, based on their respective expression values.

Based on our comprehensive transcriptome and cellular network analysis, we propose a novel protein–protein interaction network to better represent the molecular cellular response of ECs in OSA modeled by IH and epinephrine (sympathetic nervous system activation). We constructed a network comprising 45 protein nodes and 168 interaction edges, which reveal the regulatory landscape of ECs in OSA (Fig. 7).

The genes showing differential expression are mapped onto the network with respect to their expression levels. The protein nodes with no attributions are suggested as complementary network nodes which are essential for the transmission of cellular signaling under OSA induced IH. This novel network hypothesizes the complex interplay between hypoxia, epinephrine, and gene expression in HAECs, providing systems biology level insights into the cellular mechanisms of OSA. The proposed network has 3 significantly distinct subnetwork modules cytokine-mediated TNFα signaling *via* NF-κB, Wnt/LRP/DKK, and cell cycle regulation. While both IH and SH lead to pro-inflammatory signaling, the intensity and nature of the inflammation differ significantly between the two conditions, likely due to significant HIF-1α transcriptional activation in SH. The variations in cell cycle regulation and vascular endothelial responses are likely attributable to the distinct temporal patterns of hypoxia exposure, which uniquely influence cellular signaling pathways as well as differential HIF-1α transcriptional activation. NF-κB influences a broad spectrum of genes encoding proteins that regulate inflammation, including IL-1, IL-6, IL-8, TNFα, transforming growth factor β1 (TGF-β1), metalloproteinases, C–X–C motif chemokine ligands, and intercellular adhesion molecule 1 (ICAM-1). These components may exert immunomodulatory actions and contribute to the increased production of reactive oxygen species through a chronic inflammatory cycle. Each gene in the cytokine-mediated TNFα signaling might be implicated in the pathophysiology of OSA, potentially contributing to atherosclerosis or other cardiovascular diseases.^29^ The regulatory cytokine nodes in this module can be exploited in atherosclerosis models of IH *in vivo*.^30,31^ OSA is linked to vascular inflammation and the activation of endothelial cell adhesion molecules (CAMs), particularly through TNFα signaling pathways.^32^ ICAM-1, VCAM-1, and E-selectin levels correlates with BMI of OSA patients and E-selectin is independently associated with the apnea-hypopnea index.^33^ Furthermore the relationship between OSA and systemic inflammation is well acknowledged in humans and short neck dogs, notably through alterations in the levels of VEGF-A and CCL2.^34–36^

Involvement of Wnt pathway members may be attributed to EC response to IH which can be independent of OSA. DKK1 levels alterations independently associated with CVD.^37^ In cardiomyocytes, DKK-1 promotes the endocytosis of LRP5/6, disrupting GPCR signaling and exacerbating DNA damage under ischemic conditions. Conversely, IGFBP-4 associates with LRP5/6 when Wnt ligands are present, inhibiting the Wnt/β-catenin signaling pathway activation, which serves as a safeguard against ischemic injury to the myocardium.^38^ IH interferes with normal endothelial cells and can disrupt endothelial cell homeostasis, potentially leading to endothelial dysfunction. This contrasts with the effects of SH, where endothelial proliferation is a compensatory mechanism to improve oxygenation.^39^ For example, hypoxic intratumoral regions lead to the release of pro-angiogenic factors like TNFα, VEGF, and HIFs, which promote angiogenesis and suppress immune cell adhesion and activation. Whereas the EC disruption caused by IH may result in impaired vascular repair and maintenance, contributing to the pathophysiology observed in OSA, including increased cardiovascular risk and inflammation. This suggests a distinct impact of IH compared to SH on endothelial health and highlights the importance of understanding these mechanisms in the context of diseases like OSA. Endothelial senescence is a significant contributor to various cardiovascular and metabolic diseases and senescent endothelial cells and their secreted factors significantly contribute to endothelial dysfunction.^40,41^ Senescent ECs are highly active, secretory, pro-inflammatory. Cellular senescence is a cell fate where cells cease to divide which leads to the downregulation of cell cycle genes.^42^ Downregulation of mitotic cell cycle regulation genes under IH and epinephrine in cell cycle regulation subnetwork in OSA network model proposes the involvement of endothelial senescence in OSA associated vascular disorders. Further research is essential to clarify the effects of OSA on cellular senescence.^43^

## 5. Conclusions

This study shows that in contrast to SH which induces HIF-1α transcriptional response, IH does not activate HIF-1α or its transcriptional response. We also found that epinephrine is able to induce a mild increase in HIF-1α expression; however, this effect was lost under IH. Our analysis primarily focused on HIF-1α due to its well-established role in hypoxic responses. Other transcription factors may also contribute to the observed differences, as indicated by the gene regulatory network enrichment analysis. The newly constructed endothelial cell response pathways presented in this study include both HIF-1α-dependent and -independent responses to reduced oxygen levels, demonstrating a coherent adaptive response to sustained and intermittent hypoxia with the involvement of sympathetic activation. Future studies could further investigate the transcriptional regulation of downstream genes influenced by the transcription factors listed under IH conditions. Additionally, we did not detect markers typically associated with senescence or cytotoxicity in IH, suggesting that IH may not induce the same level of cellular stress as SH, which could account for the absence of cell cycle arrest markers. This study provides comprehensive biological insights into the cellular mechanisms involved. These observations are crucial for understanding the cellular dynamics under conditions that mimic OSA and could have significant implications for therapeutic strategies targeting endothelial function in diseases associated with hypoxia.

## Author contributions

Conceptualization: RCA, RBH, AGH, GMM, investigation: RCA, AYM, YT, TC, PSW, writing original draft: RCA, GMM, writing – review and editing: RCA, RBH, GMM, PSW.

## Data availability

The data supporting this article have been included as part of the ESI.† The Cytoscape network .cys file of the EC_Hypoxia_response PPI is available at: https://github.com/rengulA/EC-response-to-IH-and-SH. RNA-seq data is accessible *via* GEO (GSE205050) and (GSE279434).

## Conflicts of interest

There are no conflicts to declare.

## Supplementary Material

MO-021-D4MO00142G-s001

MO-021-D4MO00142G-s002
