# Supplementary material for: Sustained hypoxia but not intermittent hypoxia induces HIF-1α transcriptional response in human aortic endothelial cells
Source: Mol Omics. 2024 Oct 22;21(1):19–31. doi: 10.1039/d4mo00142g (PMC11563308; doi:10.1039/d4mo00142g)
Supplement: MO-021-D4MO00142G-s002 [file MO-021-D4MO00142G-s002.pdf]

Supplementary Information (SI) for Molecular Omics.
This journal is © The Royal Society of Chemistry 2024

Supporting information:

Fig S1 _ HIF-1α protein stabilization analyzed under different exposures to IH and full-size images of western blot experiments (A) that are achieved by two independent methods (**B** and **C)**

A

IH-Biorep1 (1% O_2)

30 min 1 hrs 2 hrs 3 hrs

DMOG

Normoxi

IH-Biorep2 (1% O_2)

30 min 1 hrs 2 hrs 3 hrs

HIF-1α

GAPDH

HIF-1α GAPDH

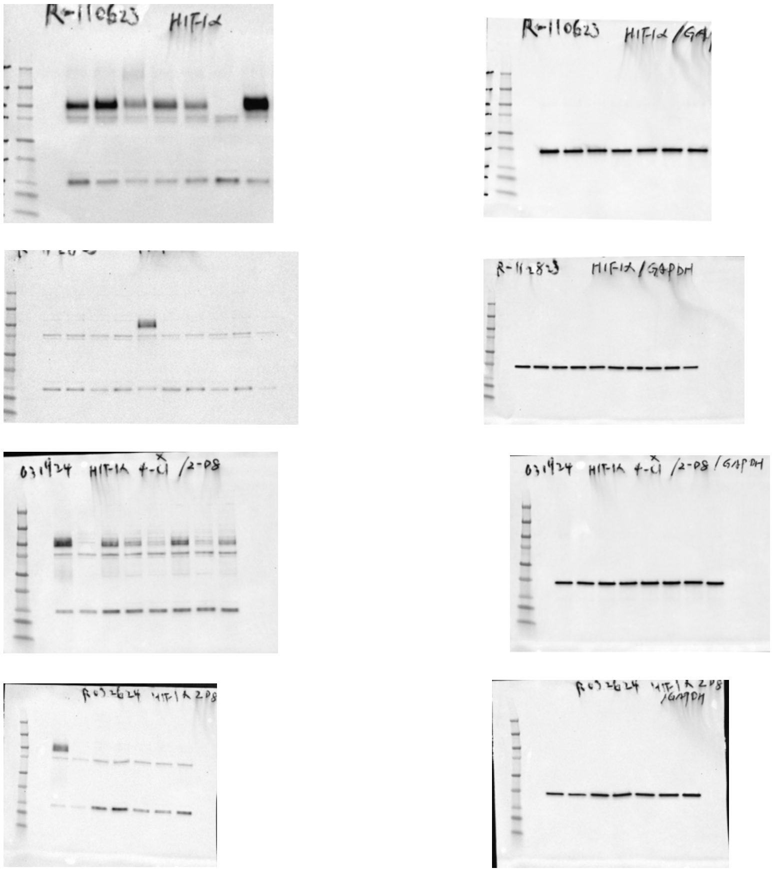

B

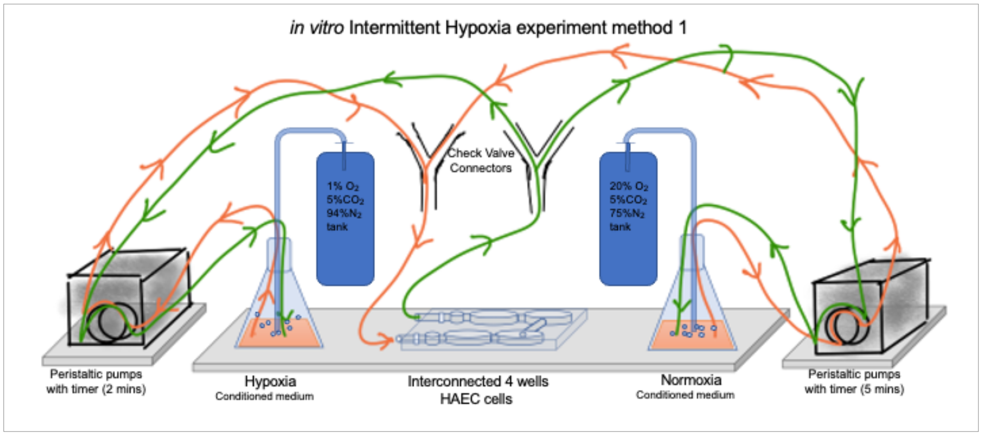
in vitro Intermittent Hypoxia experiment method 1
Check Valve Connectors
1% O2 5%CO2 94%N2 tank
20% O2 5%CO2 75%N2 tank
Peristaltic pumps with timer (2 mins)
Hypoxia Conditioned medium
Interconnected 4 wells HAEC cells
Normoxia Conditioned medium
Peristaltic pumps with timer (5 mins)

C

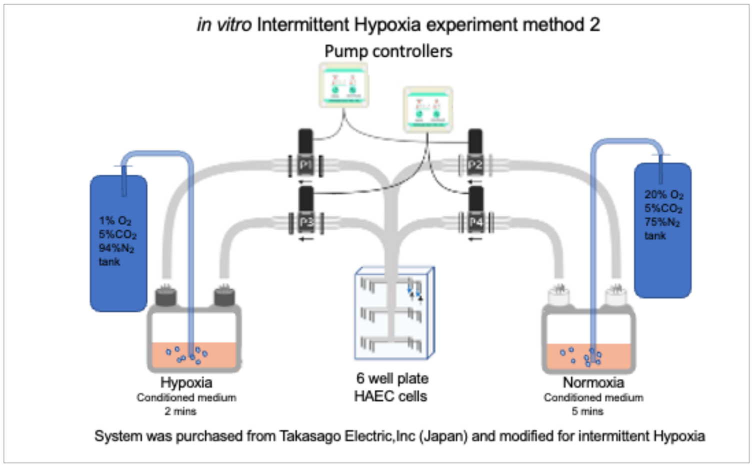
in vitro Intermittent Hypoxia experiment method 2
Pump controllers
P1
P2
P3
P4
1% O2 5%CO2 94%N2 tank
20% O2 5%CO2 75%N2 tank
Hypoxia Conditioned medium 2 mins
6 well plate HAEC cells
Normoxia Conditioned medium 5 mins
System was purchased from Takasago Electric,Inc (Japan) and modified for intermittent Hypoxia

Fig S2 _Volcano plots of Comparative gene expression in Normoxia (Ctrl), Intermittent hypoxia (IH) and sustained continuous hypoxia (CH or SA) treated with Epinephrine (Epi)

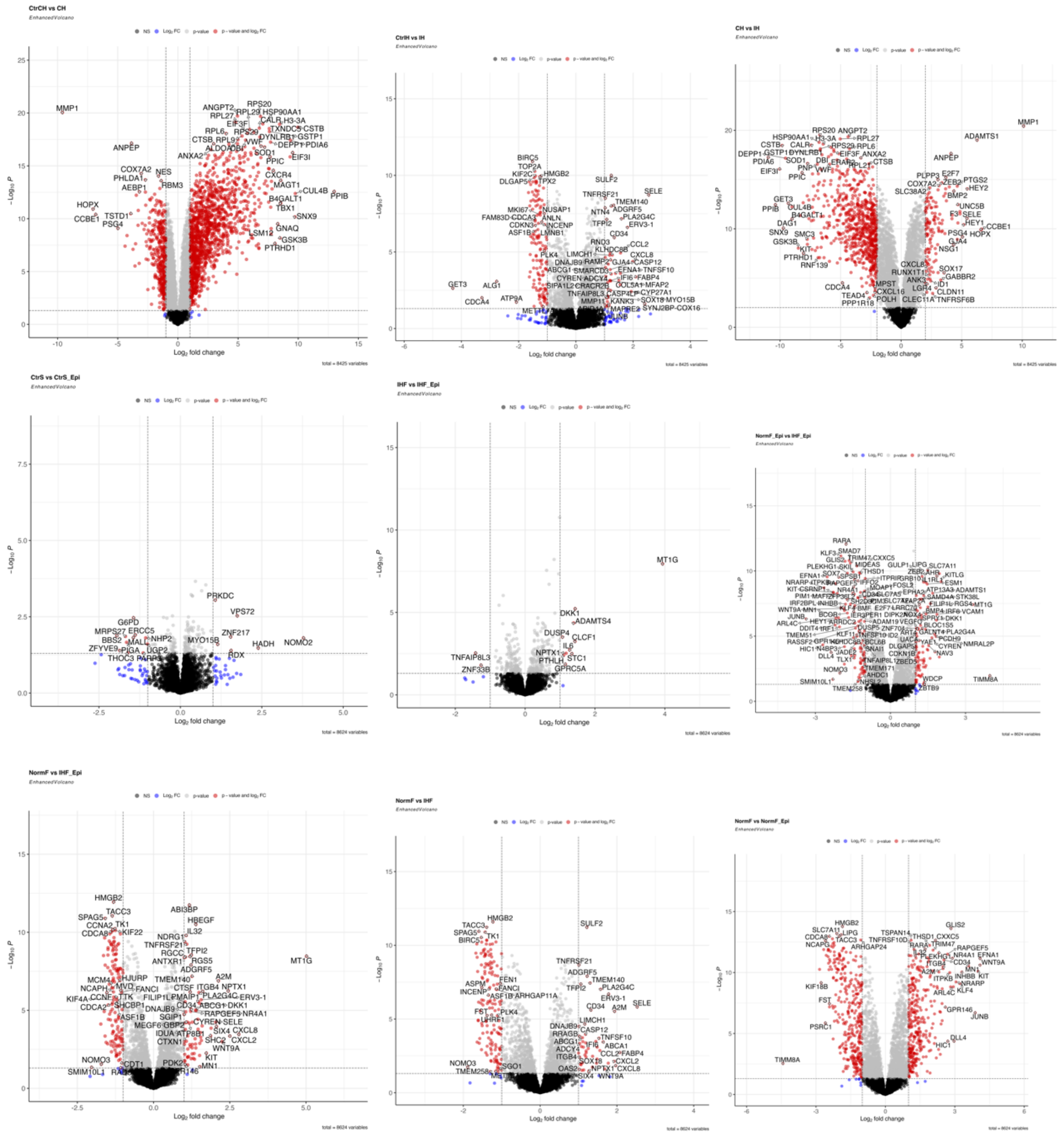

Fig S3 _ Ctrl vs Epi and Ctrl vs IH-Epi DoRothEA and Cytoscape PPI. (A) DoRothEA Transcription factor enrichment normoxia Ctrl vs Epi and Ctrl vs IH+Epi. The Cytoscape PPI with visualization of downstream gene expression profiles included the following Ctrl vs Epi, Ctrl vs IH+Epi, Ctrl vs SH and Ctrl vs IH. Upregulated genes are in red, and downregulated genes are in blue.

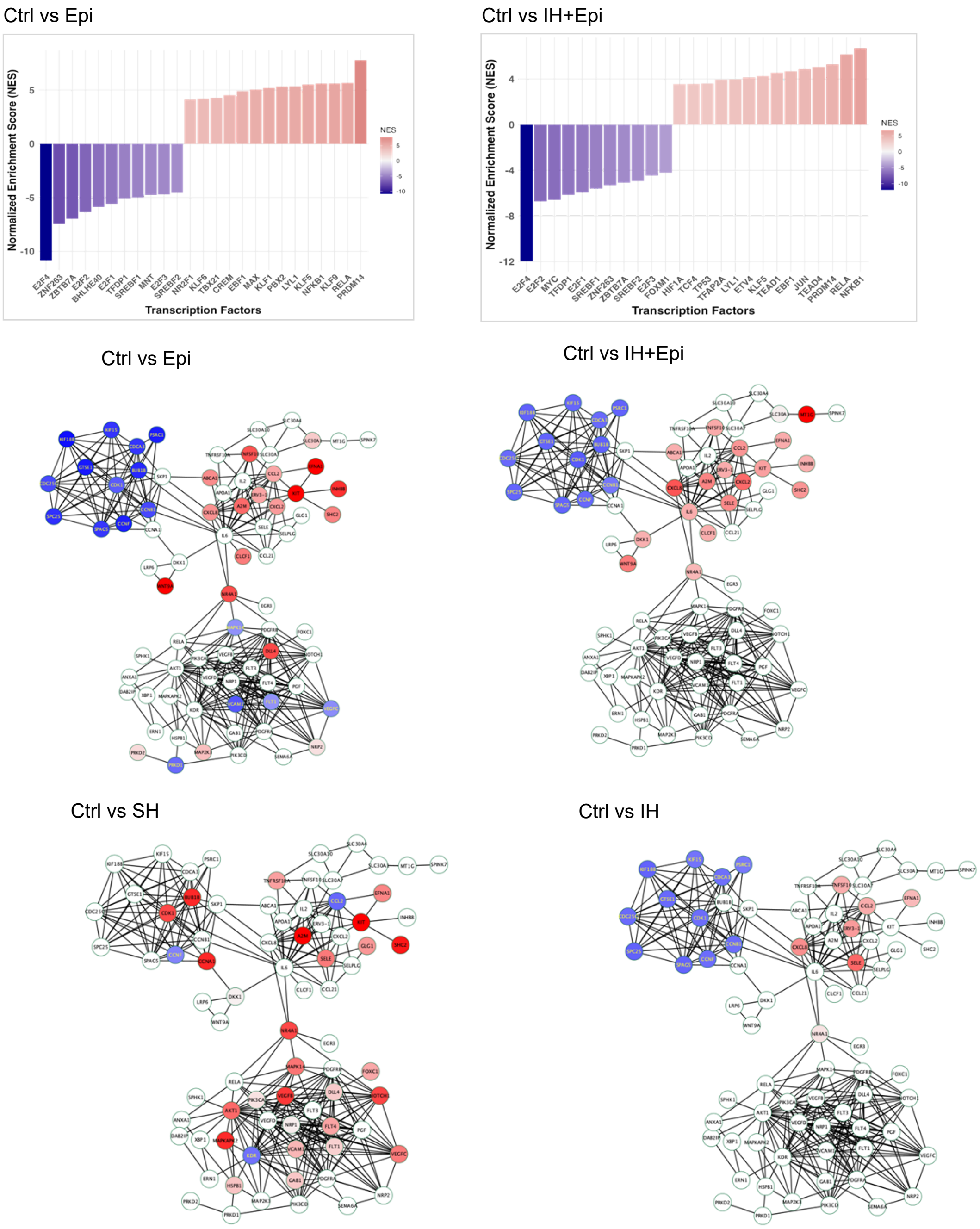

CHEA_HIF_TARGETS GENE LIST:

GPR135, HEY1, C2ORF42, CGA, WSB1, MRPL14, DYNLL1, MAFK, SEC61G, SOS1, FAM65C, P4HA2, MDGA2, BRK1, KDM4B, LONP1, DNAJB6, TMEM18, DHCR7, ELMSAN1, PPM1D, TM2D3, PNRC1, SEMA4B, INHBA, SLC6A19, PFKL, SLC9A9, BCAS4, SLC37A3, VDAC1, ALDOA, NOL3, UBA6, FOS, KLRF1, NT5DC1, SLC31A1, GOLGA1, CITED2, SERTAD2, RUNX1, TFF1, HTR5A, POU2F1, PTPRN, CCNL1, MYL4, ALDOC, ZFAT, SLC8A3, RAN, ANO6, TFRC, WISP3, MAB21L3, PARD6B, DPT, GTF2IRD2B, NAV1, PTPRM, FAM162A, ZNF331, TMEM45B, TMEM45A, BHLHE40, SCARB1, WDR60, NTHL1, GLTSCR2, RASSF7, DYRK1B, STC2, C11ORF30, TMEM183A, FBL, SLC38A2, MRPS18A, BMP4, PPP2R1A, ACOT6, PNPO, PPP1R14B, CAV1, WDR89, WDR54, CLDN14, DAAM1, PDK1, FAM13A, AKAP10, KRT80, AGPAT5, MGAT5, NUPL1, PFKP, ARRDC3, C1ORF116, SLC35E1, ZSWIM4, SLC2A1, WBSCR22, FNTB, NCOA3, PPP1R21, DHRS13, PKP2, MSANTD3, C1D, KLHDC4, TRIOBP, SLC28A1, IRX1, TRIM37, MTFP1, SPRY1, C8ORF58, ZFP36L1, 10-Mar, PRDM1, TSC22D2, UPK1B, AKAP12, MTCH2, HK2, TPD52, C11ORF49, MBTPS1, ANKZF1, CUX1, MAPK6, S100A10, KDM3A, ADM, OXSM, DSP, KDM4C, SAP30, TLE1, CRKL, PVRL4, KIAA0195, CLN5, SCD, OTUD7B, DAPK2, ALKBH5, RAI14, UBC, TMEM189, DDX50, BCL2L2, GUK1, HILPDA, ELL2, ERRFI1, EVX1, PSMD6, CECR5, DARS, INSIG2, WISP1, RIT1, SUMF1, CYP1B1, LPIN3, UBE2V2, SCYL1, SLC2A3, LDHA, GBE1, MAF, NDRG1, GRB10, SIAH2, PLOD2, NAMPT, RAPGEF6, AHNAK, FUT11, KRT19, NCOR2, GADD45B, SLC22A18AS, AKR1E2, GJA1, ITGA5, ZC3HAV1L, CDC73, N4BP3, ILVBL, RASEF, UTRN, NFKB2, QSOX1, ITFG2, RNF19A, ARID1B, ANGPTL4, ST8SIA6, C3ORF14, JMJD6, RDH11, BHLHE41, PARN, SRSF3, GPR37L1, VGLL4, EVL, TMEM88, FBXW7, GSE1, LOXL2, IGFBP3, RBPJ, PDGFB, EGFR, FEM1C, KMT2E, NUP210, CHD2, TRAM2, BZW1, UNKL, LINC00242, GPI, ELF3, DENND1A, PC, NR4A2, PPP1R13L, TRIM33, SLA, DNASE1, FOXO1, PFDN4, SPAG4, SNAPC3, MAX, DDIT4, PGAM1, ZNF19, DNM2, CLK3, FBXO42, SSUH2, ZNF572, ATP9A, PTPRB, PKM, DAB2IP, BACE2, KCTD15, ANKRD37, FAM129B, KLF10, CLIC6, RAP1GAP2, ALOXE3, MPI, RNF126, S100P, MIF, CTDSP1, NARF, PPP1R3C, PNMA2, ENO1, C1ORF27, GAPDH, FAM110C, TPCN2, COX20, LINC01011, LINC00574, BCCIP, CCNG2, ZNF407, P2RY2, IBA57, KDM5B, KXD1, BLCAP, MAML1, PFKFB3, P4HA1, LRP1, UBE2E1, TNS1, DAZL, EGLN3, ZNF395, OXSR1, VRK1, GDF15, RAD51B, EMP3, PXDN, TMEM75, NCOA5, SMC2, VKORC1, SNAPC1, MAP1LC3B, WDR5B, PFKFB4, KCNMA1, RAB6A, PPP1CB, RSBN1, MICALL2, CIART, CA9, INHA

GOBP_RESPONSE_TO_OXYGEN_LEVELS GENE LIST

msigdb/human/geneset/GOBP_RESPONSE_TO_OXYGEN_LEVELS

ABAT, ACAA2, ACE, ACVRL1, ADA, ADAM15, ADAM17, ADAM8, ADIPOQ, ADM, ADO, ADORA1, ADSL, AGER, AGTRAP, AIFM1, AJUBA, AK4, AKT1, ALAD, ALAS2, ALKBH5, ANG, ANGPT2, ANGPT4, ANGPTL4, APAF1, AQP1, AQP3, ARNT, ARNT2, ASCL2, ATF2, ATF4, ATG7, ATM, ATP6AP1, ATP6V0A2, ATP6V0D1, ATP6V1A, ATP6V1G1, ATP7A, BAD, BCL2, BECN1, BIRC2, BMP2, BMP7, BNIP2, BNIP3, BNIP3L, BRIP1, CA9, CAPN2, CASP3, CASP9, CASR, CAT, CAV1, CAV3, CBFA2T3, CBL, CBS, CCDC115, CCNA2, CD24, CD38, CDKN1A, CDKN1B, CFLAR, CHCHD2, CHRNA4, CHRNA7, CHRNB2, CIAO3, CITED2, CLCA1, CLDN3, COL1A1, CPEB1, CPEB2, CPEB4, CREBBP, CRYAB, CXCL12, CXCR4, CYB5R4, CYBB, CYGB, CYP1A1, CYSLTR1, DDAH1, DDIT4, DDR2, DIO3, DNMT3A, DPP4, DRD2, EDN1, EDNRA, EEF2K, EGLN1, EGLN2, EGLN3, EGR1, ENO1, ENSG00000274276, EP300, EPAS1, EPHA4, EPO, ERCC2, ERCC3, ERO1A, F7, FABP1, FAM162A, FAS, FGFR2, FMN2, FOS, FOSL2, FOXO1, FUNDC1, FZD4, GATA6, GNGT1, GUCY1A1, GUCY1A2, GUCY1B1, HDAC2, HIF1A, HIF3A, HILPDA, HIPK2, HK2, HMOX1, HMOX2, HP1BP3, HSD11B2, HSF1, HSP90B1, HSPG2, HYOU1, IL1A, IRAK1, ITGA2, ITPR1, ITPR2, KCNA5, KCND2, KCNJ11, KCNJ8, KCNK2, KCNK3, KCNMA1, KCNMB1, LEP, LIF, LIMD1, LMNA, LONP1, LOXL2, LPAR1, LTA, MALAT1, MAP3K7, MB, MDM2, MDM4, MECP2, MGARP, MIEF1, MIR106B, MIR126, MIR140, MIR145, MIR146A, MIR17, MIR20A, MIR21, MIR210, MIR214, MIR34A, MIR448, MIR762, MLST8, MMP14, MMP2, MPL, MT-ATP6, MT-ND4, MT-ND5, MT3, MTHFR, MTOR, MYB, MYC, MYOCD, MYOD1, NDNF, NDP, NDRG1, NDUFS2, NF1, NFE2L2, NGB, NKX3-1, NOL3, NONO, NOP53, NOS1, NOS2, NOTCH1, NOX1, NPEPPS, NPPC, NR4A2, OPRD1, OXTR, P2RX2, P2RX3, P4HB, PARP2, PCK1, PDK1, PDK3, PDLIM1, PDPN, PENK, PGF, PGK1, PHB2, PICK1, PIK3CB, PIN1, PINK1, PKLR, PLAT, PLAU, PLEKHN1, PLK3, PLOD1, PLOD2, PMAIP1, PML, POLB, POSTN, POU4F2, PPARA, PPARD, PPARG, PRKAA1, PRKCE, PSEN2, PTGIS, PTGS2, PTK2B, RAD21, RBPJ, REST, RGCC, ROCK2, RORA, RPTOR, RWDD3, RYR1, RYR2, SCAP, SCFD1, SCN2A, SDHD, SFRP1, SIRT1, SIRT2, SIRT4, SLC11A2, SLC1A1, SLC29A1, SLC2A1, SLC2A4, SLC2A8, SLC39A12, SLC6A4, SLC7A5, SLC8A3, SLC9A1, SMAD3, SMAD4, SOD2, SOD3, SOX2, SOX4, SRC, SRF, STC1, STC2, STOX1, STUB1, SUV39H1, SUV39H2, TBL2, TEK, TERC, TERT, TFRC, TGFB1, TGFB2, TGFB3, TGFBR3, TH, THBS1, TIGAR, TLR2, TM9SF4, TMBIM6, TMEM199, TNF, TP53, TREM2, TRPV4, TSC1, TWIST1, TXN2, TXNRD2, UBQLN1, UCN3, UCP2, UCP3, USF1, USP19, VASN, VCAM1, VEGFA, VEGFB, VEGFC, VEGFD, VHL, WDR83, WTIP, XRCC1, ZEB2, ZFP36L1

ENDOTHELIAL CELL RESPONSE GENE LIST

AKT1, XBP1, MAPK14, FLT3, HSPB1, DLL4, PDGFRA, DAB2IP, PDGFRB, GAB1, KDR, PIK3CA, NRP1, FLT1, VCAM1, VEGFD, VEGFB, SPHK1, MAP2K3, MAPKAPK2, ANXA1, PIK3CD, RELA, PGF, VEGFC, NOTCH1, ERN1, FOXC1, FLT4, EGR3, NRP2, PRKD1, SEMA6A, PRKD2, A2M, CCL2, IL2, APOA1, CXCL8, IL6, ABCA1, SKP1, SELE, BUB1B, CCNA1, CCNB1, SPC25, PSRC1, GTSE1, CCNF, CDC25C, KIF18B, CDCA3, KIF15, SPAG5, CDK1, ERV3-1, SELPLG, EFNA1, TNFSF10, KIT, CXCL2, CCL21, DKK1, CLCF1, NR4A1, LRP6, WNT9A, GLG1, TNFRSF10A, SLC30A7, INHBB, SHC2, MT1G, SPINK7, SLC30A1, SLC30A10, SLC30A4,
